# Longitudinal Predictors of Institutionalization in Old Age

**DOI:** 10.1371/journal.pone.0144203

**Published:** 2015-12-14

**Authors:** André Hajek, Christian Brettschneider, Carolin Lange, Tina Posselt, Birgitt Wiese, Susanne Steinmann, Siegfried Weyerer, Jochen Werle, Michael Pentzek, Angela Fuchs, Janine Stein, Tobias Luck, Horst Bickel, Edelgard Mösch, Michael Wagner, Frank Jessen, Wolfgang Maier, Martin Scherer, Steffi G. Riedel-Heller, Hans-Helmut König, AgeCoDe Study Group

**Affiliations:** 1 Department of Health Economics and Health Services Research, Hamburg Center for Health Economics, University Medical Center Hamburg-Eppendorf, Hamburg, Germany; 2 Department of Primary Medical Care, Center for Psychosocial Medicine, University Medical Center, Hamburg-Eppendorf, Hamburg, Germany; 3 WG Medical Statistics and IT-Infrastructure, Institute of General Practice, Hannover Medical School, Hannover, Germany; 4 Central Institute of Mental Health, Medical Faculty Mannheim/Heidelberg University, Mannheim, Germany; 5 Institute of General Practice, Medical Faculty, Heinrich-Heine-University Düsseldorf, Düsseldorf, Germany; 6 Institute of Social Medicine, Occupational Health and Public Health, University of Leipzig, Leipzig, Germany; 7 Department of Psychiatry, Technical University of Munich, Munich, Germany; 8 Department of Psychiatry, University of Bonn, Bonn, Germany; 9 German Center for Neurodegenerative Diseases (DZNE), Bonn, Germany; 10 Department of Psychiatry and Psychotherapy, University of Cologne, Cologne, Germany; University of Perugia, ITALY

## Abstract

**Objective:**

To investigate time-dependent predictors of institutionalization in old age using a longitudinal approach.

**Methods:**

In a representative survey of the German general population aged 75 years and older predictors of institutionalization were observed every 1.5 years over six waves. Conditional fixed-effects logistic regressions (with 201 individuals and 960 observations) were performed to estimate the effects of marital status, depression, dementia, and physical impairments (mobility, hearing and visual impairments) on the risk of admission to old-age home or nursing home. By exploiting the longitudinal data structure using panel econometric models, we were able to control for unobserved heterogeneity such as genetic predisposition and personality traits.

**Results:**

The probability of institutionalization increased significantly with occurrence of widowhood, depression, dementia, as well as walking and hearing impairments. In particular, the occurrence of widowhood (OR = 78.3), dementia (OR = 154.1) and substantial mobility impairment (OR = 36.7) were strongly associated with institutionalization.

**Conclusion:**

Findings underline the strong influence of loss of spouse as well as dementia on institutionalization. This is relevant as the number of old people (a) living alone and (b) suffering from dementia is expected to increase rapidly in the next decades. Consequently, it is supposed that the demand for institutionalization among the elderly will increase considerably. Practitioners as well as policy makers should be aware of these upcoming challenges.

## Introduction

The number and proportion of old age individuals is expected to increase sharply in developed countries in the next decades [1]. As the need for long term care is positively associated with old age, the number of individuals in need for care is expected to increase markedly [2]. Most individuals in need for care prefer to live in their homes as long as possible in order to keep their social networks and to maintain their familiar environment [3–5]. Furthermore, from a public and private perspective, admission to a nursing home (NH) may be associated with a tremendous financial burden. Therefore, predictors of institutionalization have been studied extensively in the last decades [6–10]. However, most of these studies used static models which means that they used a static set of baseline characteristics as predictors. Consequently, it is almost unknown [11–14] how *changes* in predictors influence the probability of institutionalization.

Hence, in our study we analyzed time-dependent variables assumed to be relevant for institutionalization, such as deteriorations in walking performance or losing the spouse by death or divorce. Thus, we aimed at determining factors affecting the probability of transitions to NH or old-age homes in a longitudinal setting. In particular, we analysed in our main model how marital status, depression, dementia, and physical impairments (mobility, hearing and visual impairments) affect the probability of transitions to institutionalization. This knowledge is important in order to fully understand the causality leading to admission to NH or old age-home, respectively. This, in turn, may reveal new approaches for prevention or delay of institutionalizations which is essential as institutionalization can be viewed as a “last” and very expensive option.

## Methods

### Ethics statement

The ethics committees of the participating centers approved the study (reference numbers: 050/02 (University of Bonn), 2079 (Faculty of Medicine, University of Düsseldorf), 2817/2007 (Hamburg Medical Association), 309/2007 (Faculty of Medicine, University of Leipzig), 2007-253E-MA (Medical Ethics Commission II, University of Heidelberg at the University Medical Center of Mannheim), 713/02 (Faculty of Medicine, Technical University of Munich)). The study was conducted according to the principles expressed in the Declaration of Helsinki. Written informed consent was obtained from all participants and/or from their respective guardian. Once a patient had been diagnosed with dementia, written informed consent was obtained from a proxy (e. g. spouse). Proxies provided, for example, information about basic activities of daily living of the subject if MMSE < 25.

### Sample

Data were derived from the German Study on Ageing, Cognition and Dementia in Primary Care Patients (AgeCoDe). This is a population based prospective cohort study. Individuals were recruited by General practitioners’ (GP) offices at six study centres (Leipzig, Hamburg, Dusseldorf, Mannheim, Bonn and Munich) in Germany in 2003 and 2004 which is considered as baseline. Since then, patients as well as their proxies were interviewed by trained staff every 1.5 years (follow-up wave 5: 2011/2012). Individuals were included in the cohort if they fulfilled the following criteria at baseline: (i) age ≥75, (ii) absence of dementia and (iii) at least one contact with the GP during the last 12 months. Individuals were excluded if they met at least one of the following criteria: insufficient knowledge of the German language, consultations only via home visits, residence in a NH, severe illness the GP would deem fatal within 3 months, deafness, blindness, lack of ability to provide informed consent and not being a regular patient of the participating practice. Detailed information regarding the sampling frame were published elsewhere [15]. The study has been approved by the local ethics boards of all participating centers and written informed consent was obtained from all patients.

In our analysis we draw on six waves (baseline: n = 3,031), including 260 non-institutionalized individuals at baseline who were admitted to NH or old-age home sometime after baseline. Thereby, 41 individuals were admitted to NH or old-age home by the time of follow-up wave 1; wave 2: 47 individuals; wave 3: 56 individuals; wave 4: 50 individuals; wave 5: 66 individuals). Thus, we included solely individuals who were institutionalized sometime after baseline. We also included individuals with scores of 4/5 on the Global Deterioration Scale at baseline (n = 70) as transitions to NH or old age-home also occurred in these individuals. Among these 260 individuals, main reasons for lack of follow-up data were death (n = 71) and, to a markedly lesser extent, refused participation (n = 7).

### Institutionalization

Institutionalization was defined as self-reported transition to a NH or an old-age home sometime after baseline. For individuals who died between two waves, proxy interviews were conducted with their relatives. In these interviews the last residence before death was reported.

### Independent variables in main analysis

We used the following *sociodemographic variables*: sex, family status (Ref.: married; single, widowed, divorced), age, the educational level according to the CASMIN classification which distinguishes between three levels (primary, secondary and tertiary education [16]) and having own children (no children vs. ≥1 child). It is important to note that the time constant independent variables sex, education and having own children were solely used to describe the sample.

*Depressive symptoms* were assessed with the 15-item version of the Geriatric Depression Scale [17]. We dichotomized the Geriatric Depression Scale (1 if Geriatric Depression Scale ≥ 10; 0 otherwise). Hence, we focus on transitions to severe depression.

According to the grade of *mobility* (no impairment, aggravated walking, and substantial mobility impairment/disability of walking), *hearing* (no impairment, mild hearing loss, and severe/profound hearing loss), and *visual impairment* (no impairment, mild visual impairment, and severe/profound visual impairment) the impairments were categorized, in each case with “no impairment” as reference. The Global Deterioration Scale [18] provides information with respect to *dementia stage* (1 = no impairment, 7 = severe dementia). This scale was dichotomized into dementia (Global Deterioration Scale > 3) and absence of dementia (otherwise). The proportion of missing values was negligible in all variables.

### Independent variables in additional analysis

Presence/absence of 28 chronic conditions was recorded by the GP in wave 2 upwards. Under the condition that a chronic condition was present, severity was rated by the GP on a 1 (mild) to 4 (severe) scale by the GP. We calculated a weighted count *morbidity* score, i.e. the sum of severity ratings for conditions scored as present. This was done to account for the severity of the chronic conditions.

*Social support* (wave 2 and wave 4) was quantified using the 14-item short form of the questionnaire for social support (F-SozU K-14) designed by Fydrich et al. [19]. To account for possible cognitive impairments, dichotomized items (1 = yes; 0 = no) were used. Consequently, social support was represented by the sum of 14 dichotomized items.

*Physical* and *cognitive activities* (wave 1 upwards) were captured by a measure adapted from a scale originally developed by Verghese et al. [20]. Individuals reported how often they have done sports or cognitive activities: “every day”, “several times a week”, “once a week”, “less than once a week” and “never” for each activity in the last four weeks. We created a score for physical as well as cognitive activities. For this purpose, each physical and cognitive activity was recoded in a first step: 7 = every day, 3 = several times a week, 1 = once a week, 0 = less than once a week/never. In a second step, by averaging these items the scores were formed.

*Frailty* (wave 4 upwards) was measured using the Clinical Frailty Scale (CSHA) [21], ranging from 1 (very fit) to 7 (severely frail). The *severity of dementia* symptoms was assessed by the Clinical Dementia Rating (CDR) [22] (wave 3 upwards) with scores of (normal), 0.5 (very mild dementia), 1 (mild dementia), 2 (moderate dementia) and 3 (severe dementia). We classified a score < 1 as “very mild dementia”, a score of 1 as “mild dementia” and a score of 2 or 3 as “moderate to severe dementia”. The Mini-Mental Status Exam (MMSE) [23] (baseline upwards) is a test for screening for cognitive impairment, ranging from 30 (best score) to 0 (worst score). For our purposes, presence of dementia was assumed if MMSE reached a value ≤ 24.

*Functional impairments* were operationalized as follows: Rather complex instrumental activities of daily living were captured by Instrumental Activities of Daily Living (IADL, 8 = best score, 0 = worst score) [24] (baseline upwards). Impairment in basic activities of daily living were quantified by the Barthel Index [25] (100 = best score, 0 = worst score) (wave 3 upwards). Since there is evidence [26,27] that incontinence is related to institutionalization, we examined urinary and fecal incontinence (components of the Barthel Index) in additional models.

### Statistical analysis

Conditional fixed-effects logistic regressions were used to estimate the effects of time dependent variables on institutionalization. Thus, we assume that the unobserved heterogeneity (e. g. genetic predisposition) is correlated with the explanatory variables. This leads standard random effects strategies to be inconsistent. In order to deal with heteroskedasticity and serial correlation, robust standard errors that cluster errors at the individual level were reported [28].

To test whether fixed effects models are needed or the more efficient random effects regressions can be used instead, Hausman test was performed. It tests the null hypothesis that the coefficients estimated by the random effects estimator are the same as the coefficients estimated by the consistent fixed effects estimator. We strongly rejected the null hypothesis in each model. Consequently, fixed effects regressions are the model of choice.

Since our main variables—marital status, depression, impairments in walking, hearing and vision and dementia—were measured in all waves, we thus used within-variations from all waves. Furthermore, we estimated several additional models to test the robustness of our findings. These models differ by adding predictors (morbidity; social support; frailty; physical activities; cognitive activities) or replacing variables (dementia: CDR or MMSE instead of Global Deterioration Scale; functional impairment (IADL or Barthel-Index) instead of Global Deterioration Scale). Moreover, the statistical analysis was repeated using a more strict definition of institutionalization, which excluded individuals with transitions from assisted living to NH or old-age home.

The level of significance was set at p < .05. All statistical analyses were performed using Stata 13.1 (Stata Corp., College Station, Texas).

## Results

### Sample characteristics

Since the baseline characteristics of institutionalized and non-institutionalized individuals with a different number of waves were compared elsewhere [29], we present solely the baseline characteristics of individuals who were institutionalized sometime after baseline (Table 1). These are the individuals included in conditional fixed effects logistic regressions. Mean age was 81.3 years (±3.8 years), the majority of participants was female (77.3%), widowed (57.7%) and had primary education (58.1%).

**Table 1:** Sample characteristics at baseline: Individuals who were institutionalized sometime after baseline (n = 260).

Variables	
Age: Mean (SD)	81.3 (±3.8)
Gender: N (%)	
Female	201 (77.3)
Male	59 (22.7)
Education: N (%)	
Low	151 (58.1)
Middle	80 (30.8)
High	29 (11.1)
Family status: N (%)	
Single	23 (8.8)
Married	67 (25.8)
Divorced	20 (7.7)
Widowed	150 (57.7)
Own Children: N (%)	
No	61 (23.5)

### Regression analysis

Table 2 presents the results of the conditional fixed-effects logistic regression. The probability of admissions to nursing or old-age homes significantly increased with transitions from marriage to widowhood, occurrence of severe depression, walking and severe hearing impairments, as well as dementia. Other changes in marital status and occurrence of visual impairments did not affect the dependent variable.

**Table 2:** Longitudinal predictors of institutionalization: Results of conditional fixed effects logistic regressions (Odd ratios reported).

	Independent variables	Institutionalization
**Marital status (Ref. Married)**	Single	14.54
		(3.74e-05–5.653e+06)
	Widowed	78.32**
		(5.512–1,113)
	Divorced	13.69
		(0.557–336.3)
**Depression (Ref.: Geriatric Depression Scale <10)**	Geriatric Depression Scale (≥ 10)	8.349*
		(1.201–58.05)
**Mobility impairment (Ref.: No impairment)**	Aggravated walking	5.580***
		(2.705–11.51)
	Substantial mobility impairment/disability of walking	36.69***
		(13.12–102.6)
**Visual impairment (Ref.: No impairment)**	Mild visual impairment	1.297
		(0.709–2.374)
	Severe/profound visual impairment	0.647
		(0.210–1.999)
**Hearing impairment (Ref.: No impairment)**	Mild hearing loss	2.159*
		(1.095–4.259)
	Severe/profound hearing loss	9.361**
		(2.074–42.25)
**Dementia (Ref.: Global Deterioration Scale ≤ 3)**	Global Deterioration Scale (> 3)	154.1***
		(19.72–1,204)
	Observations	960
	Number of Individuals	201
	Pseudo R^2^	0.546

Comments: 95% Confidence Intervals in parentheses.

*** p<0.001

** p<0.01

* p<0.05

+ p<0.10.

Observations with missing values were dropped (listwise deletion).

We ran separate regressions for women and men, and individuals with own children as well as childless individuals (results are not shown, but are available upon request from the authors). Compared with total sample, findings were almost the same in women and in individuals with own children. Due to small sample size, findings in men and in childless individuals are hard to interpret.

### Additional models

We checked the robustness (in terms of significance and effect sizes) of our variables of interest by comparing the main model (Table 2) with alternate models (results of model specifications are not shown, but are available upon request from the authors). In a first and second specification, we additionally controlled for morbidity or social support, leading to similar results (both predictors did not achieve statistical significance). However, these findings need to be interpreted cautiously as sample size was rather small. In a third specification, we also controlled for physical and cognitive activities–with nearly identical results. The probability of institutionalization increased considerably with less physical activities. In a fourth specification, we redid regressions with CSHA instead of impairment variables and Geriatric Depression Scale. Even though sample size was very small, becoming more fragile raised the probability of institutionalization significantly.

All analyses were repeated with CDR or MMSE instead of Global Deterioration Scale. The effect of dementia on the risk of institutionalization was insensitive to the measure of dementia (CDR, MMSE) used. Moreover, for reasons of collinearity we redid everything with functional impairment (IADL, Barthel-Index) *instead* of measures of dementia. Additionally, we estimated models with urinary and fecal incontinence (components of the Barthel Index). When the functional status (IADL, Barthel-Index) got worse, the risk of institutionalization rose significantly. However, when incontinence occurred, the risk did not increase in a significant way.

Due to the extremely high OR for dementia (see Table 2), we analyzed cognitive impairment in more detail: The occurrence of mild cognitive impairment (Global Deterioration Scale ≥ 3) was only moderately associated (OR: 6.8) with institutionalization.

Moreover, with a more strict classification of institutionalization where transitions from assisted living to NH or old-age home were not taken into account, we obtained virtually the same results. Even if we focus solely on incident dementia cases (individuals with Global Deterioration Scale < 4 at baseline), findings almost remained the same. Altogether, findings of the additional models underlined the robustness of our main model.

## Discussion

### Main findings

The risk of transitions to institutionalization increased sharply with transitions from marriage to widowhood. Moreover, the risk increased considerably with occurrence of dementia (MMSE, Global Deterioration Scale, CDR). As compared with the occurrence of dementia, it is noteworthy that the occurrence of mild cognitive impairment had a much lower impact on the risk of transitions to institutionalization. Additionally, the risk rose markedly with occurrence of walking and severe hearing impairments. Furthermore, the risk of institutionalization increased with the occurrence of severe depression in individuals.

### Previous research

Overall, our findings based on time-dependent predictors extend previous studies that used a static set of baseline characteristics as predictors. More specifically, findings regarding functional impairment [12,13,26,29–31] and dementia [26,32–34] extend previous knowledge. With regard to marital status, our findings also correspond to previous research [26,29,35,36]. For example, with data derived from the Leipzig Longitudinal Study of the Aged (LEILA 75+) Luck et al. [37] investigated institutionalization in community-dwelling subjects with incident dementia. They found that compared with being married being widowed and divorced was associated with a significant shorter time until institutionalization.

Our findings regarding mobility impairments are in line with a previous study by von Bonsdorff et al. [38]. Using a prospective cohort study with 10-year surveillance on institutionalization, they found that the risk for institutionalization was markedly higher for individuals with co-existing mobility limitations and cognitive deficits compared with individuals without limitations. Furthermore, two other prospective longitudinal cohort studies [39,40] reported strong associations between physical performance and future NH admission. This is in line with our results.

A previous study [41] has also found an association between hearing impairments and institutionalization in Australia. In contrast to Wang et al. [42], no significant effects of visual impairments on the risk of institutionalization were observed. This might be explained by the fact that they did not adjust for functional and cognitive impairments as well as depression. Excluding these factors in our regressions also leads to significant effects of visual impairments (results are not shown, but are available upon request from the authors). As already shown by Bandeen-Roche et al. [43], frailty is associated with NH entry. Findings with regard to depression are in line with McCallum et al. [27], however, the previous findings are inconsistent [44] and differ by study population and classification of depression.

### Strengths and limitations

This is one of few longitudinal studies aimed at determining factors affecting admissions to NH or old age homes. In extension to Luppa et al. [29], the present study examined the influence of time-dependent variables. Thus, insights into the causal mechanisms can be derived [45]. This might help to develop interventional strategies. A further strength of our study is that we draw on an almost representative sample for the elderly population in Germany as subjects were recruited via GP offices and almost everyone of this age bracket has GP visits. Moreover, it should be highlighted that we draw on long lasting panel data with rather short time intervals between waves.

Concerning the fact that our estimates might have been biased for reasons of panel attrition caused by time-dependent variables, we examined whether there were differences at baseline between individuals with complete follow-up data and individuals who dropped out sometime after baseline. The latter group was initially more severely cognitively (Global Deterioration Scale, MMSE) and functionally (IADL) impaired. Moreover, the latter group was older, more depressed, and had more impairments in walking, hearing and vision (results are not shown, but are available upon request from the authors). This might lead to a downward bias.

Despite the fact that model fit was decent, other factors may exist that affect the probability of admission to NH. Particularly, more research is needed to clarify the (long-term) role of social support and income. Moreover, future research is called for to disentangle the complex decision-making process regarding admissions to NH–from different perspectives.

## Conclusions

Some of these factors are inevitable in very old age, whereas other factors may be not. Therefore, our findings highlight the role of physical impairments for institutionalization. Addressing these issues in future research might open up new possibilities for delaying or preventing admission to NH in old age. This is very important as institutionalization is often seen as a “last” option.

Furthermore, the role of marital status and the occurrence of dementia should be emphasized. This is crucial as (1) the number of old people living alone [46] and (2) the number of elderly individuals with dementia[47] is expected to increase substantially in the next decades. During the same period of time, the availability of informal caregivers is likely to decrease. In sum, it is most likely that the demand for institutionalization among the elderly will increase sharply. Practitioners and policy makers should be aware of these upcoming challenges.
